# Motor segmentation: a key neuromuscular impairment in people with parkinson’s disease

**DOI:** 10.1007/s00221-025-07189-3

**Published:** 2025-11-02

**Authors:** Rebecca J. Daniels, Christopher A. Knight

**Affiliations:** Department of Kinesiology and Applied Physiology, University of Delaware, 100 Discovery Blvd , Newark, DE 19713 USA

**Keywords:** Yank, Neurodegenerative disease, Muscle, Bradykinesia

## Abstract

Healthy adults (OA) achieve rapid isometric force production with a brief, high amplitude burst of neural excitation. In some people with Parkinson’s disease (PwPD), transient reductions in neural excitation (motor segmentation) reduce rates of force development (RFD) and prolong contractions. Segmentation has strong relationships with time and rate-based measures of slowing in rapid contractions and is reliably measured from the second derivative of force (F”(t)). We sought more information about how segmentation affects neuromuscular control in PwPD. Aim 1 was to determine the prevalence of PwPD with segmentation (PD_Seg_). Aim 2 was to determine how force performance differs in PD_Seg_, PwPD without segmentation (PD_NoSeg_), and OA. Aim 3 was to quantify force segment durations. Fifty-seven PwPD ON medication and 22 OA performed rapid isometric finger abduction contractions to 20–60% of maximal voluntary contraction force. The median number of force segments to 90% of peak force were measured from F”(t) zero crossings. Additional outcomes included median times to peak force (tPF) and peak RFD (tRFD), and peak RFD (RFDpk). 68% of PwPD had segmentation (median segments ≥ 2, 95% CI [0.55 0.80]). PD_Seg_ had slower tPF, tRFD and RFDpk than PD_NoSeg_ and OA (all *p* ≤ 0.012, 0.38 ≤ *r* ≤ 0.85). PD_NoSeg_ and OA did not have statistically different tPF, tRFD, or RFDpk (*p* > 0.05). PD_Seg_ had consistent segment durations (coefficient of variation ≤ 25.5%) and shorter first segment durations compared to PD_NoSeg_ and OA (*p* < 0.001, *r* ≥ 0.68), indicating PD_Seg_ had reduced neuromuscular excitation prior to peak force. Segmentation identifies specific pathophysiology in neuromuscular control that exacerbates slowing in isometric force production.

## Introduction

A substantial body of literature on rapid movements describes robust neuromuscular rate-control mechanisms in healthy adults. For example, when healthy adults perform most-rapid isometric contractions, very high initial motor unit firing rates (Desmedt and Godaux 1977; Van Cutsem et al. 1998) result in a succinct burst in the surface electromyogram (EMG) with amplitude that scales with the rate of force development (RFD; Klass et al. 2008; Park and Stelmach 2006). Similarly, in rapid single-degree-of-freedom dynamic contractions, high velocity is achieved by a triphasic burst pattern including two agonist EMG bursts alternating with one antagonist burst. The first agonist burst amplitude determines the peak velocity (Brown and Cooke 1981; Corcos et al. 1989; Gottlieb et al. 1989; Hallett and Marsden 1979). In people with Parkinson’s disease (PwPD), researchers noted departures from these mechanisms. In some PwPD, EMG and force changes resembled exaggerated aging, where prolonged EMG bursts with reduced amplitudes resulted in reduced RFD, prolonged time to peak force, and greater variability compared to healthy older adults (OA; Daniels et al. 2024; Wierzbicka et al. 1991). In others, greater impairments were evident, including silent periods in EMG during the task (Daniels et al. 2024; Wierzbicka et al. 1991) that we call motor segmentation (segmentation). With multiple reduced-amplitude EMG bursts required to reach peak force, transient reductions in RFD introduced progressive slowing and a segmented appearance of the force-time curve (Wierzbicka et al. 1991). These findings agreed with observations in the dynamic contraction literature, where reduced first agonist burst amplitude and duration resulted in lower movement velocity (Carboncini et al. 2001; Robichaud et al. 2004) and multiple triphasic burst cycles required to complete the task (Hallett and Khoshbin 1980; Robichaud et al. 2004). Both exaggerated aging and segmentation contribute to bradykinesia, the cardinal Parkinson’s disease (PD) symptom that includes slow movements (Berardelli 2001), but they each express different manifestations of the pathophysiology. Segmentation separates the best and worst performing PwPD during isometric force tasks (Daniels et al. 2024; Wierzbicka et al. 1991) and provides significant information about the origins of symptom presentation that the current clinical assessments may not reveal.

Segmentation refers to the abnormal silent periods or transient reductions in neuromuscular excitation in PwPD during rapid contractions. Segmentation can be measured as an increased number of agonist EMG bursts (Daniels et al. 2024) or from excess zero crossings in the second-time derivative of isometric force recordings (Daniels et al. 2024; Howard et al. 2022; Stelmach et al. 1989). The multiple, short EMG bursts required to produce rapid contractions in some PwPD has been implicated as the mechanism of bradykinesia (Hallett and Khoshbin 1980), distinguish PwPD from other movement disorders (Berardelli et al. 1996) and healthy adults (Daniels et al. 2024; Vaillancourt et al. 2006; Wierzbicka et al. 1991), and are sensitive to improvements from PD treatments including exercise (David et al. 2016), levodopa medication (Baroni et al. 1984; Benecke et al. 1987; Berardelli et al. 1986; Vaillancourt et al. 2004, 2006) and deep brain stimulation (Vaillancourt et al. 2004, 2006). Measuring segmentation from isometric force recordings provides the advantages of being a reliable measure in PwPD (Howard et al. 2022), while requiring less instrumentation and easier analysis.

The prevalence of PwPD with segmentation (PD_Seg_) is currently unclear based on varied small studies that reported subgroup counts based on segmentation in force or EMG (Daniels et al. 2024; Palmer et al. 1991; Wierzbicka et al. 1991). Though other studies indicate that a majority of PwPD exhibited additional or segmented EMG bursts during at least some trials of rapid contractions (Baroni et al. 1984; Berardelli et al. 1986; Carboncini et al. 2001; Flament et al. 2003; Hallett et al. 1977; Hallett and Khoshbin 1980; Pfann et al. 2001; Teasdale et al. 1990), it is difficult to ascertain the prevalence or the extent this affects their movement without a larger sample size and formalized definition of segmentation. Informed by a prior study (Howard et al. 2022), we categorize a person as PD_Seg_ if most of their rapid force pulses have two or more segments during the excitation phase of force production. In the Howard et al. (2022) study, all ten PwPD met this criterion. Thus, Aim 1 was to estimate the prevalence of segmentation among PwPD at Hoehn and Yahr (H&Y) stages 1–3 and while on medication. Expecting that the majority of PwPD have segmentation, we hypothesized that greater than 50% of PwPD in the present sample will have segmentation.

Furthermore, direct comparisons of PD_Seg_ and PwPD without segmentation (PD_NoSeg_) are sparse, define group membership by varying criteria, and often provide the most illuminating findings through individual subject observations rather than group-level statistical comparisons (Daniels et al. 2024; Palmer et al. 1991; Wierzbicka et al. 1991). Despite these challenges, findings suggest greater slowing and meaningful differences in rate control in PD_Seg_ compared to PD_NoSeg_ (Daniels et al. 2024; Palmer et al. 1991; Wierzbicka et al. 1991). Considering the value of effectively characterizing distinct disorder in a heterogeneous disease, this warrants objective comparisons of slowing between larger subgroups of PwPD formed through well-defined criteria. Thus, Aim 2 was to compare PD_Seg_, PD_NoSeg_, and OA in measures of force initiation performance and physical function, and compare PwPD subgroups in motor symptom measures. We hypothesized that PD_Seg_ would have lower peak RFD (RFDpk), greater time to 90% peak force, and poorer physical function compared to PD_NoSeg_ and OA. We also hypothesized that PD_Seg_ would have poorer motor symptom scores compared to PD_NoSeg_.

Previous studies also commented that EMG bursts in PD_Seg_ occur at regular intervals (Robichaud et al. 2002; Wierzbicka et al. 1991); Howard et al. (2022) found low variance in first segment durations across PD_Seg_. Additionally, Robichaud et al. (2002) found that PwPD were unable to increase the duration of EMG bursts like healthy adults, even with medication. These findings suggest that regular interruption in neuromuscular excitation is characteristic of segmentation and provide an opportunity to quantify its apparent periodicity in force recordings to better understand the disorder. Thus Aim 3 was to describe the temporal characteristics of segmentation and their consistency in isometric force recordings. We hypothesized low variability in the segment durations and the periods of slowing between segments. Altogether, the broad hypothesis of this work is that segmentation measures can be linked to specific abnormalities in neural control and offer promising clinical utility.

## Materials and methods

Recruitment and data collection for this cross-sectional observational study (von Elm et al. 2007) occurred August 2022-May 2025.

### Subjects

PD inclusion criteria included a clinical PD diagnosis and age 40–90 years old. Exclusion criteria for all subjects included known orthopedic limitations, injury, disease or movement/neurological conditions other than PD that might interfere with motor function during testing, body mass index ≥ 40 and individuals without normal or corrected vision. Included OA scored ≥ 26 on the Montreal Cognitive Assessment (MoCA) 8.1 and ≤ 10 on the Movement Disorder Society-Unified Parkinson’s Disease Rating Scale part III (MDS-UPDRS-III). All subjects could walk unassisted and follow instructions. Everyone completed all study procedures. Data from both groups was collected at the University of Delaware and community centers throughout Delaware.

This study included 57 PwPD (45 males, aged 68.7 ± 7.8 years, 5.1 ± 4.0 years since diagnosis, 1.74 ± 0.10 m tall, mass: 80.3 ± 14.5 kg, H&Y stage: 2.0 ± 0.5, levodopa equivalent daily dose: 586.0 ± 450.4 mg, MDS-UPDRS-III: 34.8 ± 13.0) on their usual medications and 22 OA (11 males, aged 68.8 ± 9.7 years, 1.69 ± 0.11 m tall, 76.0 ± 15.8 kg, MDS-UPDRS-III: 3.7 ± 3.5).

## Function measures

Subjects performed two trials of the timed up and go (TUG; Podsiadlo and Richardson 1991), including a practice trial at their normal walking speed and a timed trial performed “as quickly and safely as possible” which was used for analysis. Subjects performed three trials of maximal isometric grip (Jamar Plus, Patterson Medical, Warrenville, IL, USA) in the tested hand (see below). Subjects were seated with their elbow at 90° and the greatest force was used for analysis.

## Isometric rapid force pulse protocol

**Fig. 1:**
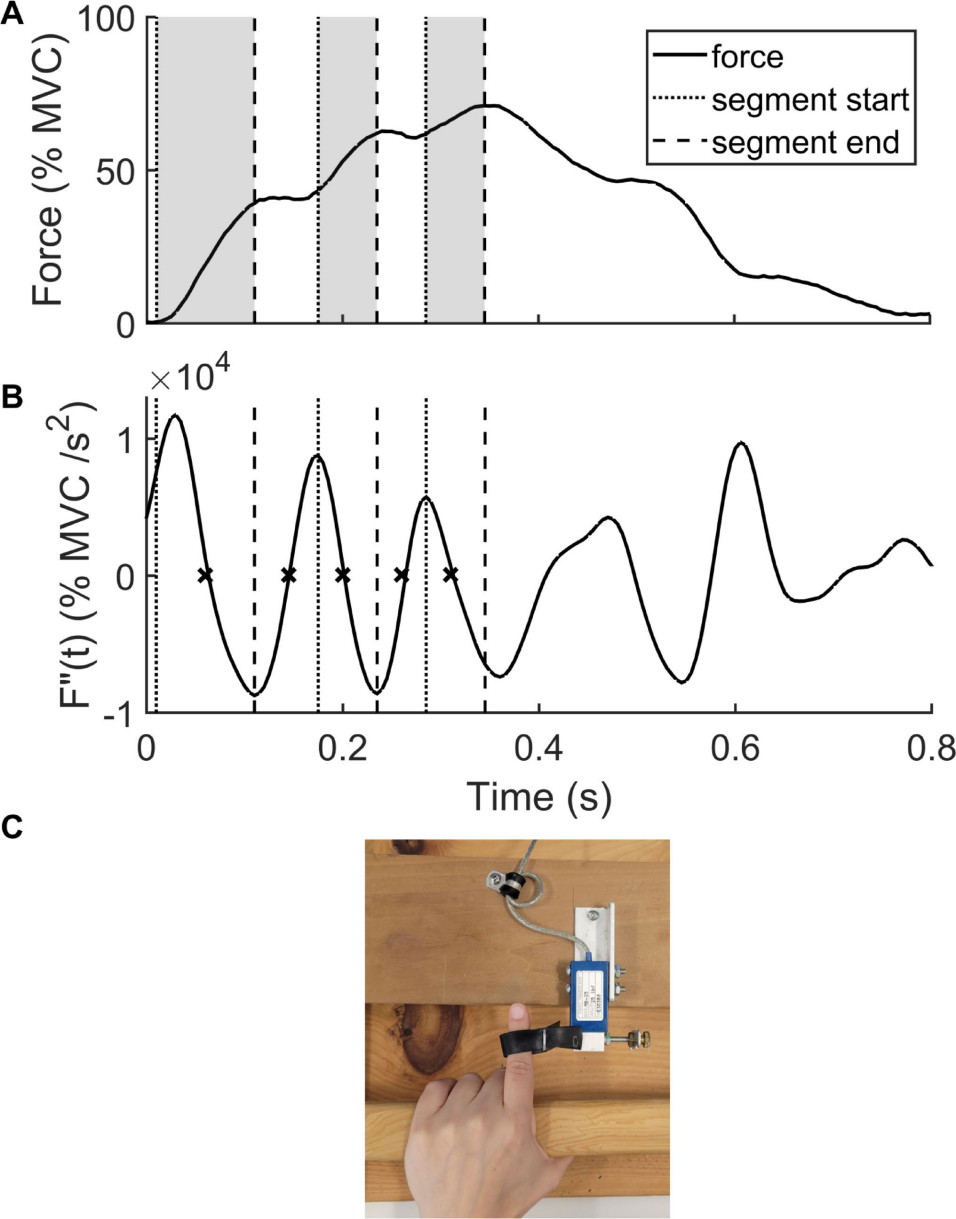
Visual depiction of segment and slowing period calculations. Top panel shows a single force pulse with three segments up to peak force. Middle panel shows the second derivative of force (F”(t)) by time. The initiation of each segment prior to peak force is indicated with a dotted line, corresponding to either force initiation or a peak in the second derivative of force between two zero crossings (each denoted by an x). The end of each segment is noted by a dashed line, which corresponds to minima in the second derivative of force between two zero crossings. The bottom panel shows the same force pulse plot, with segments indicated by shaded periods between dotted and dashed lines. Slowing periods are considered to be the plateaued regions between shaded segments. MVC = maximal voluntary contraction force

PD subjects sat at a standard height table with their shoulder flexed approximately 20°, their elbow flexed approximately 110°, with their self-reported more symptomatic hand resting on a custom force measuring device described by Howard et al. (2022). This corresponded to 31 PwPD using their dominant hand. If PwPD did not currently have a more symptomatic side, we tested the side where they first noticed symptoms. The hand OA used was randomized, which corresponded to 14 OA using their dominant hand. Subjects viewed feedback of their force on a computer monitor. Subjects grasped a wooden support bar parallel to the table and an elastic strap fastened their extended index finger to a rigid bumper attached to a force transducer (SM-50, Interface Inc., Scottsdale, AZ, USA) that measured their index finger abduction force produced in the transverse plane (Fig. 1C).

Force was amplified, low-pass filtered (50 Hz), and sampled at 200 Hz (NI cDAQ-9178, Module 9329, National Instruments, Austin, TX, USA). DASYlab software (Measurement Computing Corporation, Norton, MA, USA) controlled the digitization and provided real-time visual force feedback. Subjects performed three maximal voluntary isometric index finger abduction contractions (MVC), with one minute of rest between trials. The greatest MVC was used for analysis. Force feedback was normalized to the greatest MVC on a vertical bar graph in %MVC units.

Subjects performed five 60 s trials of rapid isometric finger abduction force pulses in three different amplitude ranges that were indicated by the investigator (20–40%MVC, 40–60%MVC, and 60–80%MVC). Subjects were instructed to produce fast pulses within amplitude ranges rather than to hit a specific target, to promote the subject’s emphasis on speed rather than accuracy (Gordon and Ghez 1987). The present study was focused on rapid force pulses approximating 40%MVC, but the overall protocol included the wider range of amplitudes to address other aims not reported here. A beep cued subjects to perform pulses approximately 3–5 s apart with variability in timing to prevent anticipation. Subjects performed a final MVC afterwards to test for fatigue. To reduce potential bias, all subjects were given standardized instructions and the force analysis was algorithm-driven. The data collection and analysis methods were the same for both groups.

Matlab software (Mathworks, Natick, MA, USA) was used to analyze force data. First, the peak force of each pulse was identified. We calculated the first (RFD) and second time-derivatives of force (F”(t) using a moving slope (D’Errico 2025) with an overlapping 50 ms window (Howard et al. 2022). The 50-ms window was chosen based on pilot work that supported its appropriate sensitivity to fluctuations in force with the temporal features of segmented force output in Parkinson’s disease. A RFD threshold of 20% MVC/s determined the start and end of each pulse, which an investigator visually inspected and confirmed for each pulse. The time to peak force was determined from the time of force initiation to peak force. Additionally, the time from force initiation to 90% peak force (F90) was calculated to quantify slowing during the excitation phase of the pulse (Howard et al. 2022). The F90-based approach is based on the premise that impaired pulse performance should be analyzed in three phases; (1) the excitation/ rising phase of the force pulse, (2) the switching or transition phase from excitation to relaxation, and (3) the relaxation phase. Difficulties in each of these phases can have an impact on total pulse duration with the possibility that different neuromuscular processes are involved in each. Additional force dependent measures included time to RFDpk, RFDpk in the overall force pulse and in the first segment, first segment duration and the segment where RFDpk occurred.

The number of segments was calculated by adding one to the number of zero crossings in F”(t), and dividing the total by two (Howard et al. 2022). Segments from force initiation to F90 (segments to F90) and to peak force were counted for each pulse. From a varied number of pulses within each subject, 41 were randomly selected between 20 and 60% MVC. This range of force levels allowed us to include a sufficient and equal number of pulses in the proximity of 40% MVC, knowing that subjects were instructed to focus on speed rather than the accuracy of pulse amplitude (Howard et al. 2022). The odd number of pulses (41) avoids median values including 0.5, which do not represent real observations (i.e. there are no half segments). Subjects were classified as having segmentation if their median number of segments to F90 was ≥ 2. For all force measures, medians were computed from the randomly-selected pulses for each subject to be used for analysis.

Segment durations during the active phases of force production from force initiation to peak force, as well as the periods of reduced RFD between segments (slowing periods) were calculated as follows. The end of each force segment was the minimum between zero crossings within F”(t). The start of each force segment (excluding the first which begins at force initiation) was the maximum between successive zero crossings. For example, the first segment would occur between force initiation and the minimum between the first two zero crossings within the pulse. The second segment would occur between the maximum between zero crossings two and three, and the minimum between zero crossings three and four. Slowing periods are the regions between the end of the previous segment and the beginning of the next segment (see Fig. 1 for a visual depiction).

## Clinical symptom ratings

A trained investigator evaluated ratings of each subject’s motor symptoms on their usual medications using the MDS-UPDRS-III (Goetz et al. 2008). The Kinesia ONE (Heldman et al. 2014) system was simultaneously used to quantify ratings of speed while performing upper extremity bradykinesia MDS-UPDRS-III tasks for 15 s to reduce potential rater bias since bradykinesia is usually rated with a greater bias towards amplitude (Heldman et al. 2011). Additionally, Kinesia ONE was used to quantify measures of resting, kinetic and postural tremor. All symptoms were rated on a 0–4 scale with 0 indicating no evidence of the symptom, and 4 indicating a very severe symptom. A bradykinesia subscore of Kinesia ONE speed was the sum of the finger taps, hand movements, and pronation-supination speed ratings of the side tested during the isometric force protocol.

### Statistical analysis

SPSS version 29 (IBM, Armonk, NY) was used to perform all statistical tests with the significance level set to *p* < 0.05. An exact binomial test with Clopper-Pearson 95% confidence interval was used to determine whether the prevalence of PD_Seg_ exceeds 50%. Descriptive characteristics were compared between groups using a one-way ANOVA, and between PD groups using independent t tests. Pre and post MVCs were compared using a paired t test within each group. We compared peak force levels across groups using a one-way ANOVA. The Shapiro-Wilk’s test determined that distributions of additional force measures did not meet normality assumptions. Thus, Kruskall Wallis tests were used to compare groups in force measures, TUG, and grip strength. When appropriate, pairwise comparisons were performed with p values adjusted with a Bonferroni correction for multiple comparisons. Z scores from the pairwise comparisons were converted to *r* to estimate effect size according to Field (2013). Effect sizes were categorized as small (0.10), moderate (0.30), or large (0.50) according to Cohen (1988). Symptom scores were compared between PD subgroups using Mann-Whitney U tests. Medians, interquartile ranges, and coefficients of variation (CV) were calculated for the timing of the first five segments and subsequent slowing periods in PD_Seg_. Friedman’s ANOVA was used to compare durations across the five segments and slowing periods, and stepwise step-down follow up analyses were used to determine homogenous subsets.

## Results

### Prevalence of segmentation

**Fig. 2:**
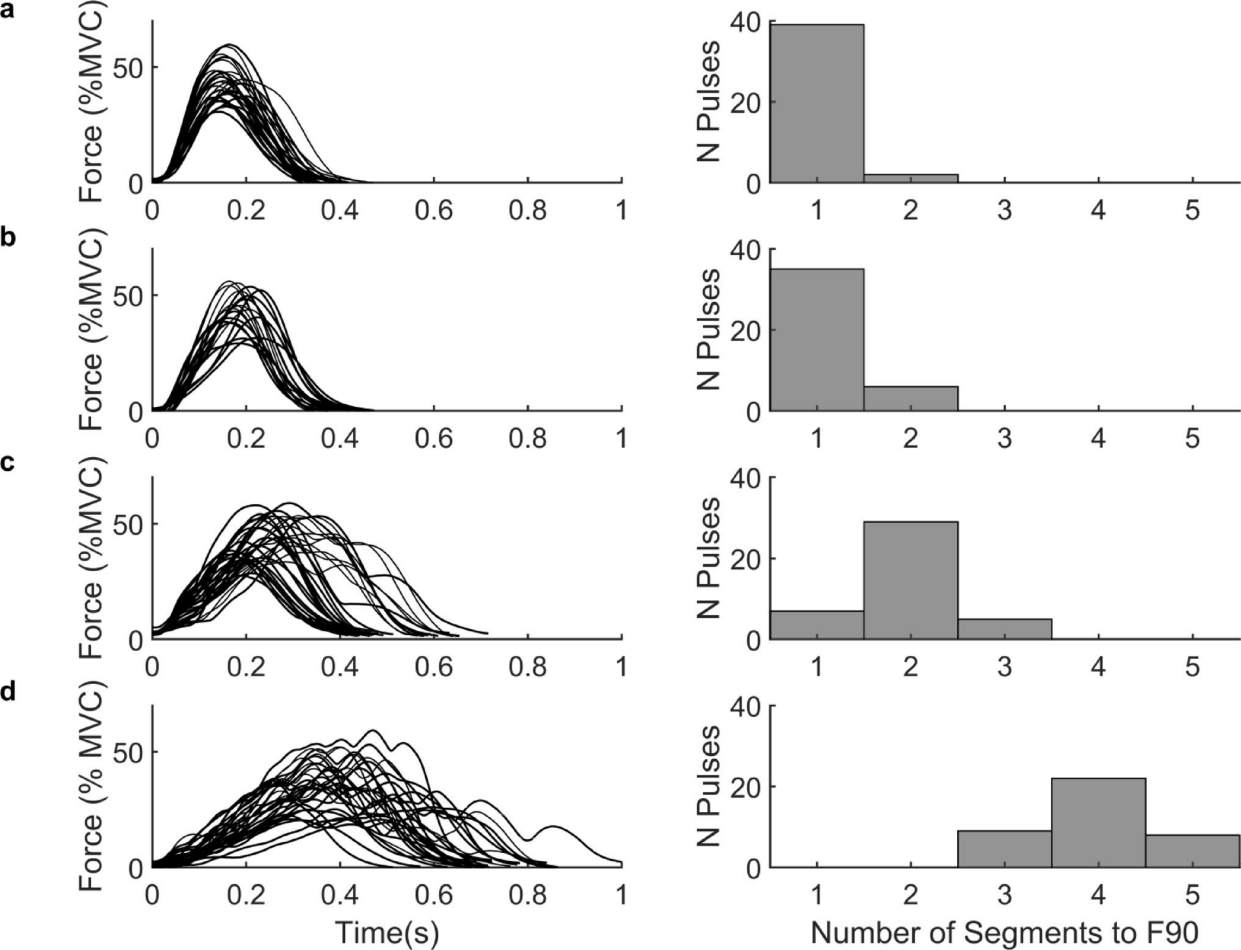
Representative pulses and segmentation histograms for a subject within each group. (**a**) Representative subject for the older adult group, (**b**) a person with Parkinson’s disease without motor segmentation, (**c**) a person with Parkinson’s disease with motor segmentation, and (**d**) a person with Parkinson’s disease with a median of 4 segments to F90. From left to right, figures depict 40 randomly selected force pulses from the subject aligned by force initiation, and the number of segments observed up to 90% of peak force (F90) for each of those force pulses in a histogram. From panel **a** to panel **d**, subject characteristics were as follows: **a**) 81 years old, MDS-UPDRS-III score of 3; **b**) 68 years old, MDS-UPDRS-III score of 20, 0.7 years since diagnosis, Hoehn-Yahr stage 2, levodopa equivalent daily dose: 450 mg; **c**) 64 years old, MDS-UPDRS-III score of 34, 4.3 years since diagnosis, Hoehn-Yahr stage 2, levodopa equivalent daily dose: 450 mg; **d**) 62 years old, MDS-UPDRS-III score of 35, 3.0 years since diagnosis, Hoehn-Yahr stage 2, levodopa equivalent daily dose: 225 mg. MVC = maximal voluntary contraction force, N = number

**Fig. 3:**
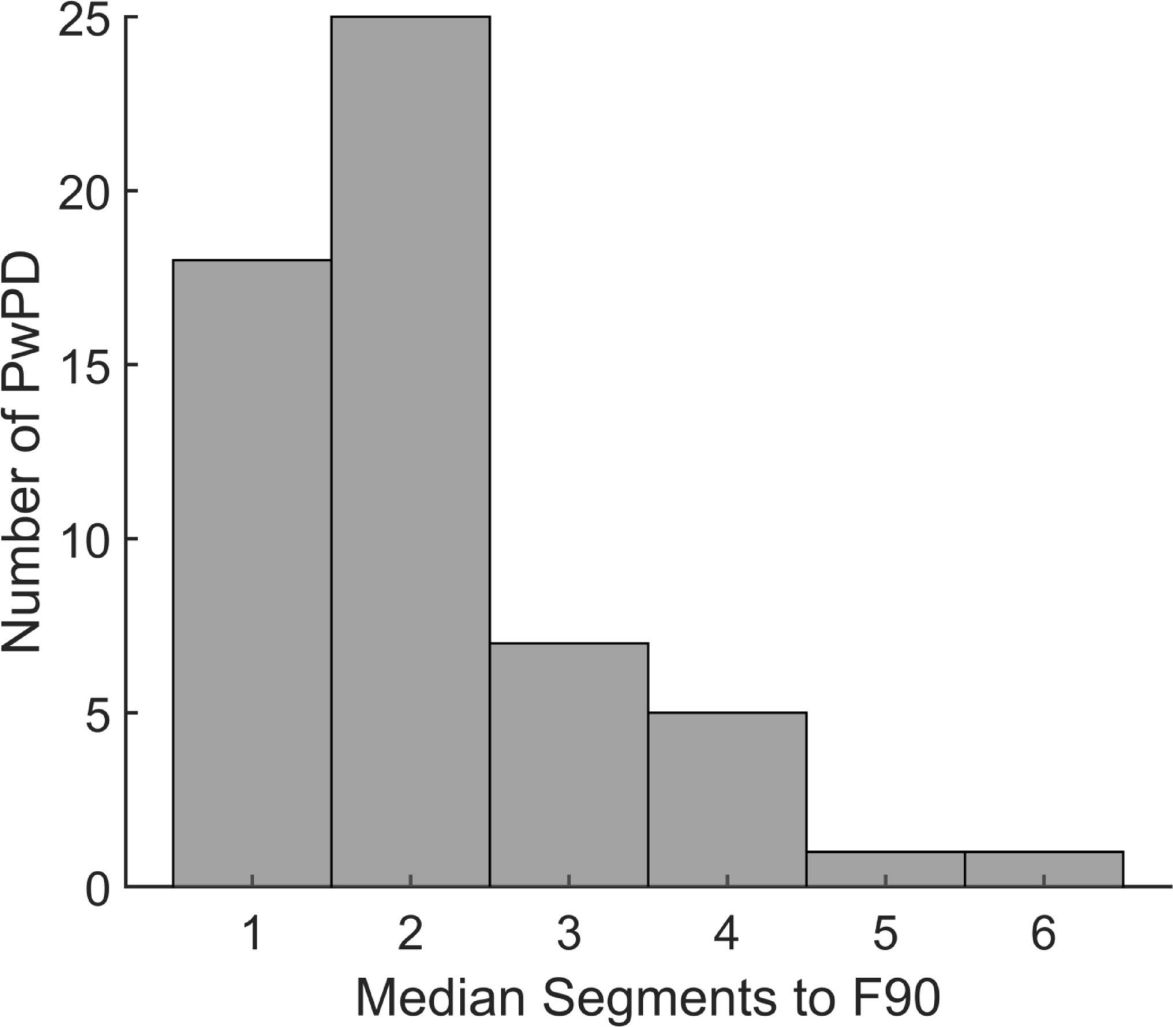
Median number of segments up to 90% of peak force (F90) for each person with Parkinson’s disease (PwPD)

Representative pulses and segmentation histograms are plotted for an OA, a PD_NoSeg_, a PD_Seg_, and a PD_Seg_ with more severe segmentation (Fig. 2). 39 of 57 PwPD (68%) had segmentation based on a median segmentation count to F90 ≥ 2. The prevalence was significantly greater than the hypothesized 50% (*p* = 0.008, 95% CI [0.55 0.80]). Figure 3 shows a histogram of each PwPD’s median segments to F90.

## Group differences

Groups were not different in age (F(2,76) = 0.004, *p* = 0.996), height (F(2,76) = 2.03, *p* = 0.138) or weight (F(2,76) = 0.723, *p* = 0.488). PD subgroups were not different in levodopa equivalent daily dose (*t*(55) = 1.42, *p* = 0.160) or time since medication intake in levodopa users (*t*(52) = 0.85, *p* = 0.401), but PD_Seg_ were diagnosed 3.4 years earlier (*t*(55) = 3.25, *p* < 0.001).

Subjects were not fatigued evidenced by increased post MVC values compared to pre MVC values in OA (*t*(21) = 2.50, *p* = 0.021), PD_NoSeg_ (*t*(17) = 2.43, *p* = 0.027), and PD_Seg_ (*t*(38) = 2.31, *p* = 0.026). Peak force was not different between groups (F(2,75) = 1.48, *p* = 0.234) which facilitated further comparisons. Peak force mean ± standard deviation was 41.3 ± 3.2%MVC for OA, 41.5 ± 4.2%MVC for PD_NoSeg_, and 40.0 ± 3.5%MVC for PD_Seg_.

**Fig. 4:**
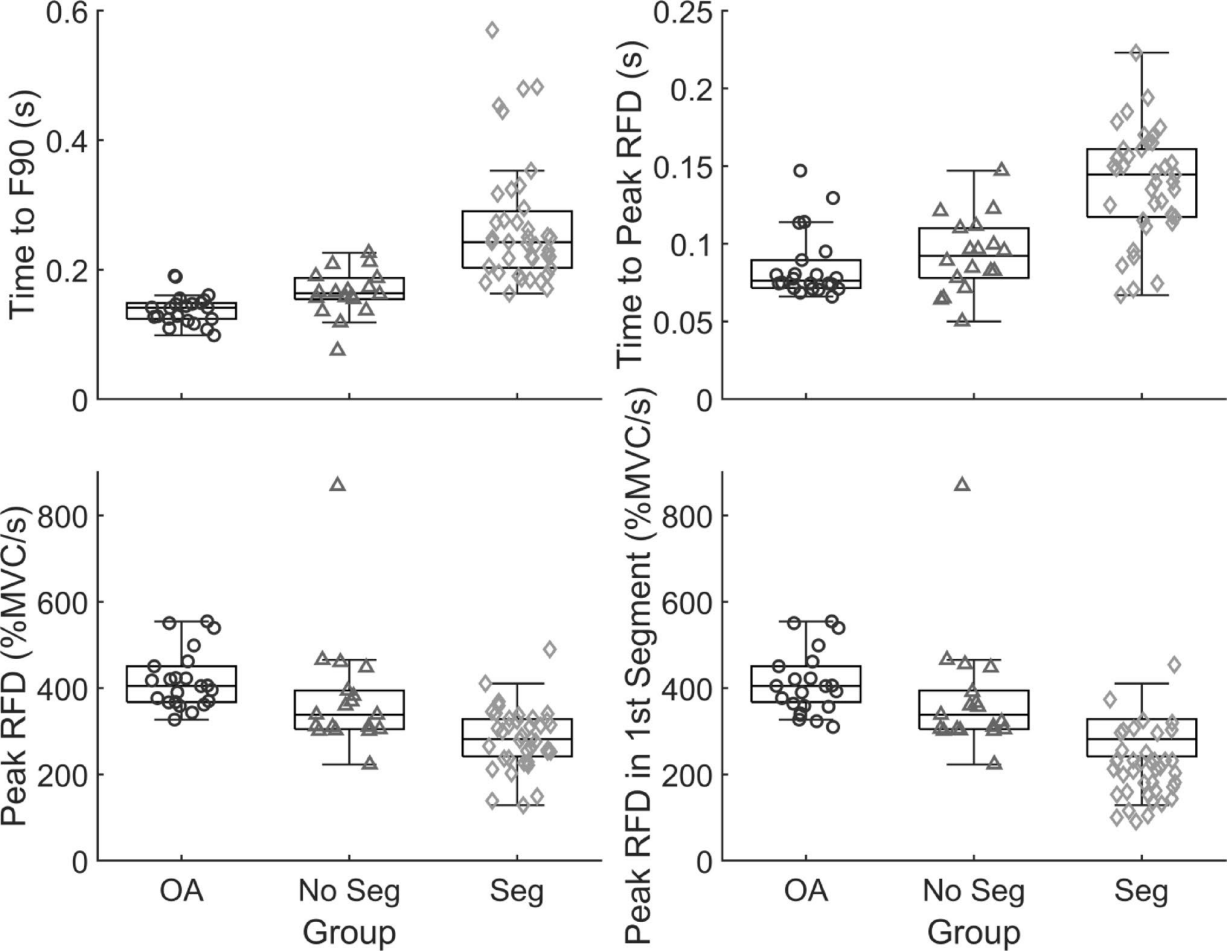
Group box plots with individual subject medians overlaid for force initiation variables. Boxplots include older adult (OA), people with Parkinson’s disease without motor segmentation (No Seg) and people with Parkinson’s disease with motor segmentation (Seg) groups for time to 90% of peak force (F90, top left), time to peak rate of force development (RFD, top right), peak RFD in % maximal voluntary contraction per second units (%MVC/s, bottom left), and peak RFD within the first force segment (bottom right)

The median ± interquartile range for the percent of pulses with ≥ 2 segments in each group was 4.2 ± 7.9% in OA, 27.4 ± 15.7% in PD_NoSeg_, and 82.0 ± 25.4% in PD_Seg_. Table 1 shows differences in force and function measures between OA, PD_NoSeg_, and PD_Seg_. Table 2 shows results of pairwise comparisons and effect sizes. Figure 4 shows boxplots for force initiation variables.

**Table 1:** Group comparisons for force and clinical measures

Measure	People with segmentation median (IQR)	People without segmentation median (IQR)	Healthy Older adults median (IQR)	*H*(2)		*p*
Number of segments to F90	2 (1)	1 (0)	1 (0)	69.8	< 0.001	
Number of segments to peak force	2 (1)	1 (0)	1 (0)	64.1	< 0.001	
Peak RFD (% MVC/s)	281.9 (86.8)	338.2 (89.2)	405.2 (82.9)	35.8	< 0.001	
Time to F90 (s)	0.243 (0.088)	0.164 (0.033)	0.141 (0.025)	52.1	< 0.001	
Time to peak force (s)	0.303 (0.135)	0.201 (0.045)	0.172 (0.036)	47.4	< 0.001	
1st Segment duration (s)	0.086 (0.019)	0.165 (0.040)	0.152 (0.046)	46.1	< 0.001	
Time of peak RFD (s)	0.145 (0.044)	0.092 (0.032)	0.076 (0.018)	33.6	< 0.001	
Peak RFD in the first segment	212.4 (94.1)	330.0 (86.7)	398.5 (93.7)	47.3	< 0.001	
Segment where peak RFD occurs	2 (1)	1 (0)	1 (0)	28.8	< 0.001	
TUG (s)	7.50 (1.77)	6.31 (1.81)	5.85 (1.20)	15.3	< 0.001	
Grip strength (kg)	38.2 (16.7)	38.9 (11.3)	31.9 (17.6)	1.1	0.567	
Kinesia one bradykinesia Speed score	3.7 (2.1)	2.5 (1.4)	2.1 (1.0)			
Kinesia one resting tremor	0.2 (0.4)	0.2 (0.6)	0.1 (0.2)			
Kinesia one postural tremor	0.2 (0.6)	0.1 (0.4)	0.0 (0.2)			
Kinesia one kinetic tremor	1.5 (0.4)	1.5 (0.4)	1.3 (0.3)			
Total UPDRS score	38 (15)	29 (19)	3 (5)			
Hoehn and Yahr stage	2 (0)	2 (0)	0 (0)			

*H*(2) = Kruskall Wallis test statistic (degrees of freedom), IQR = interquartile range, F90 = 90% peak force, RFD = rate of force development, MVC = maximal voluntary contraction force, TUG = timed up and go time, UPDRS = Movement Disorder Society-Unified Parkinson’s Disease Rating Scale Part III total score

**Table 2:** Pairwise comparisons between the groups with large effect sizes indicated in bold

Measure	People with segmentation vs. People without segmentation z, *p*, *r*	People with segmentation vs. Healthy older adults z, *p*, *r*	People without segmentation vs. Healthy older adults z, *p*, *r*
Number of segments to F90	6.60, < 0.001, 0.87	7.05, < 0.001, 0.91	0.00, 1.00, 0.0
Number of segments to peak force	6.00, < 0.001, 0.80	7.02, < 0.001, 0.90	0.51, 1.00, 0.08
Peak RFD (% MVC/s)	2.88, 0.012, 0.38	6.12, < 0.001, 0.78	2.38, 0.052, 0.38
Time to F90 (s)	4.38, < 0.001, 0.58	6.87, < 0.001, 0.88	1.84, 0.198, 0.29
Time to peak force (s)	4.02, < 0.001, 0.53	6.62, < 0.001, 0.85	1.95, 0.152, 0.31
1st Segment duration (s)	5.74, < 0.001, 0.76	5.35, < 0.001, 0.69	0.65, 1.00, 0.10
Time to peak RFD (s)	3.97, < 0.001, 0.53	5.32, < 0.001, 0.68	0.90, 1.00, 0.14
Segment where peak RFD occurs	4.24, < 0.001, 0.56	4.53, < 0.001, 0.58	0.00, 1.00, 0.00
Peak RFD in the first segment	4.31, < 0.001, 0.57	6.50, < 0.001, 0.83	1.60, 0.335, 0.25
TUG (s)	1.87, 0.184, 0.25	3.86, < 0.001, 0.49	1.57, 0.353, 0.25

F90 = 90% peak force, RFD = rate of force development, MVC = maximal voluntary contraction force, TUG = timed up and go time

PD_Seg_ had greater MDS-UPDRS-III scores (*U* = 506.0, z = 2.66, *p* = 0.008, *r* = 0.35) and slower bradykinesia speed ratings (*U* = 494.0, z = 2.46, *p* = 0.014, *r* = 0.33) than PD_NoSeg_, but were not different in H&Y stage (*U* = 387.5, z = 0.95, *p* = 0.344, *r* = 0.13), resting tremor scores (*U* = 388.5, z = 0.65, *p* = 0.515, *r* = 0.09), kinetic tremor scores (*U* = 404.5, z = 0.93, *p* = 0.352, *r* = 0.12) or postural tremor scores (*U* = 410.0, z = 1.035, *p* = 0.300, *r* = 0.14).

### Segment and slowing period durations

**Fig. 5:**
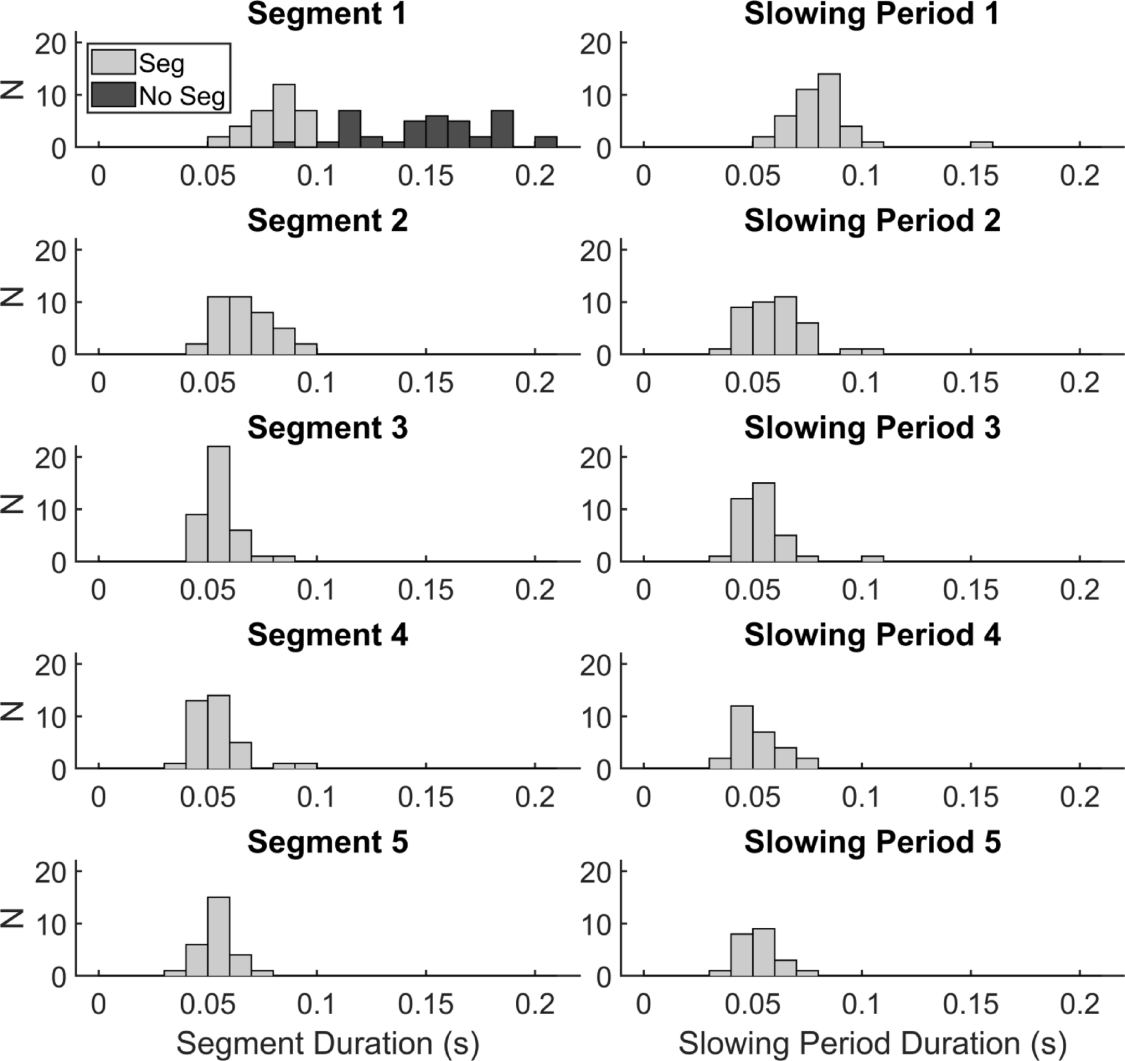
Histograms of median segment durations and slowing period durations. The median for each person with Parkinson’s disease is plotted, so that N indicates the number of subjects with values within the bin. See Fig. 1 for a visual depiction of how each duration is calculated

Figure 5 shows histograms for segment and slowing period durations. Medians ± interquartile ranges for the first five segment durations in PD_Seg_were 86.0 ± 18.9 ms (CV = 25.5%, *n* = 39), 65.0 ± 16.5 ms (CV = 18.5%, *n* = 39), 52.5 ± 9.0 ms (CV = 16.1%, *n* = 39), 53.7 ± 11.2 ms (CV = 20.6%, *n* = 35), and 52.5 ± 9.0 ms (CV = 16.3%, *n* = 27). The first five slowing period durations were 80.0 ± 12.9 ms (CV = 19.9%, *n* = 39), 59.7 ± 15.4 ms (CV = 21.6%, *n* = 39), 50.5 ± 12.6 ms (CV = 22.0%, *n* = 35), 49.0 ± 12.0 ms (CV = 18.7%, *n* = 27), and 50.1 ± 8.5 ms (CV = 16.3%, *n* = 22), respectively.

Segment durations were significantly different (χ^2^(4) = 66.06, *p* < 0.001). Step-down follow up analysis indicated that segment 1 and segment 2 durations were significantly different from all other segments, and segment 3–5 durations were within a homogenous subset. Slowing periods were also significantly different (χ^2^(4) = 51.03, *p* < 0.001), where the first silent period was longer than all successive periods, and homogenous subsets included slowing periods 2–4 and 3–5.

## Discussion

This study of motor segmentation includes the largest sample size to date, with aims to (1) identify the prevalence of segmentation in PwPD, (2) compare measures of force performance, physical function, and motor symptoms among PD_Seg_, PD_NoSeg_, and OA, and (3) describe the temporal characteristics of segmentation. In this sample of 57 PwPD tested on medication, the prevalence of segmentation was 68%. Individuals classified as PD_Seg_ exhibited greater slowing in isometric force production compared to PD_NoSeg_ and OA with large effect sizes. Across individual PD_Seg_, the duration of segments and slowing periods were remarkably consistent, indicating an abnormality in neural drive with relatively little variance. We believe these findings support further development of segmentation as a measure of neuromuscular function in PwPD. Additionally, they indicate that segmentation effectively identifies subtypes of slowing in isometric force production, including slowing similar to exaggerated aging, and disordered slowing.

Previous studies identified impairments in neuromuscular function in PwPD during rapid contractions, including insufficient initial EMG (Pfann et al. 2001; Vaillancourt et al. 2004, 2006), abnormal numbers of EMG bursts (Daniels et al. 2024; Hallett et al. 1977; Hallett and Khoshbin 1980; Pfann et al. 2001; Vaillancourt et al. 2006; Wierzbicka et al. 1991), reduced RFD (Daniels et al. 2024; Howard et al. 2022; Park and Stelmach 2007; Stelmach and Worrincham 1988) or velocity (Hallett and Khoshbin 1980; Pfann et al. 2001; Vaillancourt et al. 2004, 2006), and prolonged time to reach peak force (Daniels et al. 2024; Howard et al. 2022; Park and Stelmach 2007; Stelmach and Worrincham 1988) or displacement (Benecke et al. 1987). Hallett and Khoshbin (1980) interpreted the initial characteristics, increased variability and abnormal bursting patterns of EMG as a mechanism of bradykinesia, which distinguish PwPD from other movement disorders (Berardelli et al. 1996) and normal aging (Daniels et al. 2024; Pfann et al. 2001; Robichaud et al. 2009; Vaillancourt et al. 2004, 2006; Wierzbicka et al. 1991). EMG measures including first agonist burst duration and amplitude as well as the number of EMG bursts required to perform rapid contractions have also been used to assess the effects of exercise training (David et al. 2016), rapid movement practice (Flament et al. 2003), medication (Baroni et al. 1984; Benecke et al. 1987; Berardelli et al. 1986; Robichaud et al. 2002, 2004; Vaillancourt et al. 2004, 2006), and deep brain stimulation (Vaillancourt et al. 2004, 2006). Additionally, Robichaud et al. (2009) determined that variability in initial EMG burst duration identified PwPD with high sensitivity. While these EMG studies are informative, segmentation analysis based on isometric force recordings provides an attractive alternative because it can objectively identify reductions in neural excitation with simpler instrumentation, shorter collection times, and good reliability (Howard et al. 2022). These qualities facilitated our collection of force recordings in 57 PwPD in the laboratory and with portable instruments at community centers, with < 15 min testing for the force pulse protocol. Data analysis involving automated segment determination with visual inspection required 5–10 min per subject.

In our sample of PwPD H&Y stage 1–3, 68% had segmentation, indicated by a median number of segments to F90 ≥ 2. The larger sample size and formal definition of segmentation in this study allowed us to estimate the prevalence. To date, prevalence has varied across smaller studies (*n* = 8–24) using different upper extremity tasks. An average of 81% of PwPD across studies exhibited segmentation or additional agonist EMG bursts in at least some attempts (Baroni et al. 1984; Berardelli et al. 1986; Carboncini et al. 2001; Daniels et al. 2024; Flament et al. 2003; Hallett et al. 1977; Hallett and Khoshbin 1980; Howard et al. 2022; Palmer et al. 1991; Pfann et al. 2001; Teasdale et al. 1990; Wierzbicka et al. 1991). Other studies noted segmented EMG bursts without reporting the number of PwPD exhibiting this abnormality (Alberts et al. 2000; Berardelli et al. 1984; David et al. 2016; Robichaud et al. 2002, 2004; Vaillancourt et al. 2006). Defining segmentation as the median attempts requiring ≥ 2 segments is a more conservative metric than most previous studies, which reduced our estimate. Though the median does not capture the higher within-subject variability in PwPD, it does identify those with impairment. Additionally, withholding medication would likely increase prevalence, since levodopa reduces the number of agonist EMG bursts during rapid contractions (Benecke et al. 1987; Vaillancourt et al. 2004, 2006). Furthermore, segmentation may vary across joints, as PD does not cause identical impairments simultaneously (Pfann et al. 2001; Monje et al. 2021).

The comparison of PwPD subgroups shows that the presence of at least one period of transient slowing before peak force significantly impaired isometric force production (Fig. 2). While the PD_NoSeg_ (Fig. 2b) exhibited similar performance to the OA (Fig. 2a), the PwPD with a median of two segments (Fig. 2c) had noticeably prolonged time to peak force and a more irregular force-time curve. The three subjects exemplifying group medians (Fig. 2a-c) contrasted with the more severe case (Fig. 2d). Overall, impaired force production is clearly indicated by very prolonged time to F90 in PD_Seg_ compared to PD_NoSeg_ and OA (Table 2). While PD_NoSeg_ only exhibited trends towards slowing compared to OA, segmentation in PD_Seg_ produced large effect sizes and supports classifying these patients as having *bradykinesia with segmentation*. Segmentation also marks the severity of motor impairment, as the case with a median of 4 segments took twice as long to achieve peak force as OA and PD_NoSeg_ (Fig. 2). These findings indicate segmentation is sensitive to detect a specific abnormality in neuromuscular control, even in medicated PwPD classified as H&Y stage ≤ 3.

The presence of segmentation differentiated PwPD from OA in all force measures, with larger effect sizes (Table 2) and greater differences in Fig. 4. PD_NoSeg_ had no significant differences with OA in force measures. Meanwhile, PD_Seg_ differed from OA in every force measure and with large effect sizes. Furthermore, PD_Seg_ had large significant differences in times to F90 and peak force compared to PD_NoSeg_, similar to previous findings (Daniels et al. 2024). Wierzbicka et al. (1991) also noted that their PD_NoSeg_ reached peak force quickly with one EMG burst, while PD_Seg_ had more than doubled time to peak force and additional bursts.

In comparisons of the PwPD subgroups, differences in first segment duration and RFD illustrate specific details of this impairment. While PD_NoSeg_ achieved peak force with a single 165 ms segment (OA in 152 ms), motor output was interrupted at roughly half this duration in PD_Seg_ (Table 1; Fig. 5), indicating insufficient neuromuscular excitation to complete the task in one segment. A longer first segment duration allowed PD_NoSeg_ to reach peak force without interruption, like OA. PD_Seg_ also had lower RFDpk in the first segment and required longer times to reach RFDpk, usually in the second segment (Table 1; Fig. 4). In PD_NoSeg_, RFDpk in the first segment was nearly identical to overall RFDpk (Fig. 4), since they achieved it in the first segment in most attempts, and at similar times as OA. The one PD_NoSeg_ whose first segment duration overlapped most with PD_Seg_ could reach peak force within that time due to having the highest RFD (see outlier in Fig. 4). Though still significantly different, effect sizes were smaller when comparing RFDpk from the whole pulse to the first segment within PwPD subgroups. This can be seen as a small shift upwards in overall RFDpk in PD_Seg_ that produced the lowest RFD in the first segment (Fig. 4). Since RFD correlates with EMG amplitude (Folland et al. 2014; Josephson and Knight 2019), this suggests that initial EMG amplitude decreases prior to the end of the first segment in PD_Seg_. This agrees with EMG findings during rapid dynamic movements, where PwPD who require multiple EMG bursts to reach peak displacement can have reduced EMG amplitude in the first compared to the second burst (Pfann et al. 2001).

Clinical characteristics between PwPD subgroups were less different, with no difference in H&Y stage, and both groups including stages 1–3. This agrees with findings that RFD in the hand does not differ between earlier and later H&Y stages, though RFD is reduced in the ankle at later stages (Chung et al. 2023). Similarly, subgroups identified by Wierzbicka et al. (1991) also included H-Y stages 1–3. Likewise, TUG times were not statistically different between groups. However, PD_Seg_ had worse MDS-UPDRS-III scores and bradykinesia speed scores with moderate effect sizes, despite only a small range across the cohort. These differences support our hypothesis that segmentation contributes to disease related slowing. No differences in tremor scores also suggests that segmentation is distinct from clinically-measured tremor. The greater differences in force measures compared to clinical tests may point to specificity of testing, as subgroups were determined from force performance rather than clinical characteristics. Force measures may be better suited to study rate-control as subjects focus on producing force quickly with 3–5 s between attempts. Meanwhile, bradykinesia tasks were performed continuously over 15 s, and PwPD were instructed to perform large and fast movements, which may cause some to prioritize size over rate.

Even though segmentation increases variability in general force measures (time to peak force CV = 40.8% in PD_Seg_ vs. 26.3% in PD_NoSeg_), there is notable consistency across subjects in its temporal characteristics. Segment and slowing period durations in PD_Seg_ (Fig. 5), show little variability across subjects (CV ≤ 25.5%). Similarly, others found that more severe PwPD had the least ability to modulate the first agonist EMG burst duration (Robichaud et al. 2009) and amplitude (Pfann et al. 2001). However, the initial segment and slowing period are longer than successive durations. We note that the longer first segment duration is likely because it was calculated from the force initiation threshold rather than the F”(t) peaks used for subsequent durations (Fig. 1). Despite this, it was still reduced compared to OA and PD_NoSeg_, aligning with reports of short EMG burst durations or segments (Flament et al. 2003; Howard et al. 2022). It also coincides with findings that the first agonist burst is usually < 90 ms in segmented EMG (Carboncini et al. 2001; Farley et al. 2004; Flament et al. 2003; Robichaud et al. 2002; Vaillancourt et al. 2006).

The first slowing period has the greatest duration (80 ms). After the second, durations approximate 50 ms. Perhaps this relates to bradykinesia, where initial EMG is insufficient to reach high RFD (Wierzbicka et al. 1991) or velocity (Hallett and Khoshbin 1980). Although interruptions in the first agonist burst are counterproductive, healthy adults also use silent periods in EMG to increase RFD during rapid contractions. For example, when healthy adults produce rapid contractions from a sustained contraction, brief reductions in EMG sometimes occur just prior, lasting ~ 15–100 ms (Van Cutsem and Duchateau 2005), and result in higher motor unit firing rates and RFDpk compared to contractions lacking a silent period (Van Cutsem and Duchateau 2005; Walter 1989). In segmentation, as EMG declines prior to the end of a segment (Daniels et al. 2024), the force plateaus (Fig. 1) or declines prior to the next burst. This extended EMG reduction may provide a nervous system that cannot produce sufficiently long EMG the opportunity to increase the second burst amplitude. Palmer et al. (1991) found that when PwPD performed rapid movements following sustained contraction, all exhibited premotor silent periods during some trials. The percentage of trials exhibiting silent periods moderately correlated with peak acceleration (Palmer et al. 1991). The idea that the first extended slowing period may provide some benefit is supported by our finding that PD_Seg_ most often reach RFDpk in the second segment (Table 1), and have greater RFDpk overall than in the first segment. Additionally, the second segment had a longer duration than successive segments. Regardless, PD_Seg_ still had reduced RFDpk compared to PD_NoSeg_ (Table 2), and the prolonged slowing period may not adequately compensate when it adds to overall slowing. Palmer et al. (1991) found the incidence of segmented EMG bursts correlated with reduced acceleration regardless.

Aside from the first two segments and first slowing period, subsequent durations and slowing periods are remarkably consistent (Fig. 5) and agree with EMG findings. For example, Flament et al. (2003) noted ~ 50 ms burst durations during rapid elbow flexion in PwPD, similar to our median segment durations, and reduced from the ≥ 70 ms bursts in healthy adults (Berardelli et al. 1996). Wierzbicka et al. (1991) reported EMG bursts in segmentation at ~ 10 Hz, while Robichaud et al. (2002) described ~ 9 Hz and insensitive to medication. With the sum of segment duration and slowing period medians around 0.11 s, our findings are consistent. The last two segment durations and three silent periods also represent the most severe impairment (*n* = 22–35), as not all PD_Seg_ produced pulses with > 3 segments.

The temporal characteristics of segmentation invite comparisons with action tremor, and others have suggested the segmented EMG patterns are a form of it (Carboncini et al. 2001; Pfann et al. 2001; Robichaud et al. 2002; Vaillancourt et al. 2004). However, these connections are challenged by varying operational definitions and methods. Action tremor generally refers to postural or kinetic tremor (Louis et al. 2001), assessed by clinician ratings (Louis et al. 2001), accelerometry (Sturman et al. 2004), or spectral analysis of force (Howard et al. 2022; Vaillancourt et al. 2001), or EMG (Vaillancourt and Newell 2000). Thus far, clinical assessments of resting (Hallett et al. 1977; Hallett and Khoshbin 1980; Pfann et al. 2001) or action tremor (Pfann et al. 2001) lack notable overlap with EMG segmentation. Howard et al. (2022) also found little coexistence of segmentation and isometric force tremor. Furthermore, Vaillancourt et al. (2006) did not find treatment effects differing by tremor status, though treatment improved velocity by increasing first burst amplitude and duration, and reducing total EMG bursts. Indeed, we found no subgroup differences in clinical tremor measures, though tremor is sometimes detected in EMG before it is noticeable in clinical assessments (Pfann et al. 2001). Interestingly, three PwPD with bilateral deep brain stimulation (DBS) of the subthalamic nucleus all had a median of 2–4 segments to F90 despite DBS effectively treating postural tremor (Krack et al. 2003; Sturman et al. 2004). Similarly, Vaillancourt et al. (2006) found DBS and medication did not reduce the number of EMG bursts during rapid contractions sufficiently to match controls. Though more direct comparisons of segmentation and action tremor would clarify the relationship, it seems unlikely they reflect the same pathophysiology.

There are a few important limitations. First, the findings in this study are only applicable to the ON medication state, which reflects the interactions between Parkinsonian pathophysiology and exogenous dopamine replacement (levodopa). It is unknown if findings would be comparable in the OFF medication state. Nevertheless, despite drug effects, observations of segmentation were robust and group differences had large effect sizes. We also did not control for variations in treatment regimens or levodopa timing. Instead, participants were tested when they felt able to perform daily activities. Supporting group comparisons, there were no differences in levodopa equivalent daily dose or time since medication. Additionally, we cannot demonstrate that there were no differences in cognition between OA and PwPD groups. Whereas normal cognitive function was an inclusion criterion for OA, MoCA scores were not obtained from all PwPD. In PwPD with MoCA scores, 26/29 scored ≥ 26 (mean = 26.7). Reported associations exist between mild cognitive impairment and hand motor function (Li et al. 2024). However, our rapid force pulse task is relatively simple. All PwPD responded to instructions to produce force quickly during practice trials and communicated normally with investigators. To prevent memory lapses, subjects were reminded to focus on speed rather than accuracy following breaks. We were also unable to assess potential sex differences (Iwaki et al. 2021), due to PD_NoSeg_ only including 3 females. Finally, these results apply specifically to medicated PwPD in H&Y stages 1–3.

In summary, the segmentation protocol provides measures of disrupted force production that describe a specific impairment in neuromuscular excitation. The observed prevalence of segmentation among PwPD was 68%. PD_Seg_ exhibit slowing during rapid isometric contractions with large effect sizes compared to PD_NoSeg_ and OA. Furthermore, consistent timings of segment durations and slowing periods indicate a specific pathology that bears remarkable consistency across individuals. Segmentation provides unique information about PD pathology that is reliable, easy to obtain, and distinguishes and the best and poorest performers among PwPD.

## Data Availability

The data that support the findings of this study are available upon reasonable request.
